# Association Between Dietary Habit Changes and COVID-19 Prophylaxis During the Pandemic Among Japanese Maintenance Hemodialysis Patients

**DOI:** 10.7759/cureus.75489

**Published:** 2024-12-10

**Authors:** Toru Inoue, Tadasuke Ando, Tomochika Murakami, Shiho Hirakawa, Yoshitsugu Fujita, Toshitaka Shin, Hiromitsu Mimata

**Affiliations:** 1 Urology, Faculty of Medicine, Oita University, Yufu, JPN; 2 Organ Transplantation Promotion Project, Oita University, Yufu, JPN; 3 Hemodialysis Center, Nankai Medical Center, Saiki, JPN; 4 Faculty of Medicine, Oita University, Yufu, JPN

**Keywords:** covid-19, eating in, eating out, eating takeout, maintenance hemodialysis

## Abstract

Background: Following COVID-19, dietary habits have been altered frequently along with other societal lifestyle modifications. However, changes in the dietary habits of maintenance hemodialysis patients (MHPs) before and during COVID-19 have not been investigated.

Methods: A total of 132 MHPs were assessed for changes in their dietary habits before and during the pandemic and their association with COVID-19 prevention. Logistic regression models were used to calculate the adjusted odds ratios (ORs) with 95% confidence intervals (CIs) for the risk of COVID-19. A multivariate logistic regression analysis was performed.

Results: Approximately 27% (36 of 132) of the MHPs modified their dietary habits. Following COVID-19, the frequency of eating out decreased, and that of eating in increased significantly for dinner. However, there was no change in dietary habits for breakfast and lunch. Multivariate analysis revealed an inverse correlation between the number of eating takeout and COVID-19; that is, more eating of takeout was associated with a lower risk of contracting COVID-19.

Conclusions: Comparing before and after the pandemic, there was a shift from eating out to eating in for dinner. However, the frequency of eating takeout played a role in preventing COVID-19, suggesting that the person preparing the meal may be a more important factor than where the meal is eaten when the main route of infection is household transmission.

## Introduction

COVID-19 spread worldwide as an unknown infectious disease and was declared a pandemic by the World Health Organization (WHO) on March 11, 2020 [1]. In Japan, the government issued voluntary restrictions on going out to avoid the 3Cs (closed spaces, crowded places, and close contact) as a measure to prevent COVID-19 [2].

Subsequently, the confirmation of the fact that the main mode of transmission is aerosol, the progress made in research into prevention, the development of effective vaccines, the expansion of vaccination, and the development of therapeutic drugs and treatment strategies have allowed certain medical achievements and disease control to be achieved [3,4]. WHO announced on May 5, 2023, that it will end its declaration of a Public Health Emergency of International Concern for novel coronavirus infections [5].

After May 8, 2023, the legal treatment of COVID-19 in Japan has changed [6]. As a result, the obligation to refrain from leaving home under the law regarding COVID-19 no longer applied, and infection control measures were left to the discretion of the individual. Eating is very important for maintenance hemodialysis patients (MHPs) in terms of managing various electrolytes, body weight, fluids, and nutrition [7]. Therefore, in addition to food ingredients, seasonings, and caloric intake, where and with whom one eats, that is, dietary habits such as eating in, eating out, and eating takeout are also very important in terms of COVID-19 control [8-10]. The dietary habits of MHPs in Japan are unknown. In addition, it is unknown whether the dietary habits of MHPs changed before and during the pandemic due to the government-imposed voluntary restriction on going out and whether there is a relationship between changes in dietary habits and the risk of COVID-19 morbidity. In this study, we aimed to investigate the changes in dietary habits of MHPs before and during the pandemic and determine whether there was a correlation between changes in dietary habits and COVID-19.

## Materials and methods

Study design

This single-center longitudinal cohort study included all adult MHPs at Nankai Medical Center between August 1, 2019, and May 7, 2023. The purpose of this study was to determine the association between changes in dietary habits and COVID-19 prophylaxis during the pandemic in MHPs. 

This study was conducted in accordance with the Declaration of Helsinki and good clinician guidelines. It received approval from the Ethics Committee of Nankai Medical Center (study number: NC-R3-006).

Participants

Patients aged 20 years and older who were receiving maintenance hemodialysis (HD) three times a week from August 2019 to August 2022 at Nankai Medical Center and were in stable condition were eligible to participate in this study. Eligibility required that patients provide informed consent and complete the questionnaire. Those unable to take oral medications or complete the questionnaire and those hospitalized for a disease requiring medical treatment were excluded from the study. Data regarding patient characteristics, such as age, sex, duration of HD, medical history (including diabetes), vaccination status, and COVID-19, were collected from medical records. Dietary habits were classified into the following three categories, as previously reported: eating food prepared by yourself or your family at home or a facility was defined as eating in; eating food prepared by a restaurant or market at home or a facility was defined as eating takeout, and eating food prepared by a restaurant or market in a restaurant or market was defined as eating out [11-14].

The dietary habit survey was administered by asking respondents to indicate the approximate frequency of their breakfast, lunch, and dinner habits (eating in, eating takeout, and eating out) per week on August 1, 2019 (before COVID-19) and on August 1, 2022 (during COVID-19). Expert nurses, clinical engineers, and urologists guided MHPs who needed help completing the questionnaire. 

Before and during the pandemic, blood samples were collected immediately before HD and during fasting. White blood cell counts, C-reactive protein, total cholesterol, high-density lipoprotein cholesterol (HDL-C), low-density lipoprotein cholesterol, triglyceride, potassium (K), and phosphorus (P) levels were evaluated. 

After administering a dietary habits survey and blood sample collection, patients were followed up until May 7, 2023, to assess for contracting COVID-19. COVID-19 was defined based on positivity in a PCR test. Information on COVID-19 vaccination history was obtained from the medical charts on the day of administering the survey on dietary habits.

Statistical analysis

Categorical variables were compared using Fisher’s exact test, and continuous variables were compared using Student’s t-test or the Mann-Whitney U test. Logistic regression models were used to calculate the adjusted odds ratios (ORs) with 95% confidence intervals (CIs) for the risk of contracting COVID-19. Multivariate logistic regression analyses were performed to adjust for the appropriate number of clinically important confounders. All statistical analyses were performed using EZR (Saitama Medical Center, Jichi Medical University), a GUI for R (The R Foundation) for Statistical Computing version 2.13.0 [15]. It is an improved version of R commander (version 1.6-3), equipped with statistical functions frequently used in biostatistics [15]. The statistical significance level was set at p < 0.05.

## Results

This study included 80 males and 52 females with a mean age of 71.3±11.4 years and a mean HD duration of 107.3±84.2 months (Table 1). Before COVID-19, the frequency of eating in, eating takeout, and eating out per week was 16.25±6.0, 3.16±5.1, and 0.79±1.82, respectively (Table 2).

**Table 1:** Characteristics of the overall participants HD: Hemodialysis

Variable	Value
Male / Female	80 / 52
Age (years）	71.3±11.4
Duration on HD (months)	107.3±84.2
Patients with diabetes	58
Number of COVID-19 vaccination doses	2.89±0.57
Number of people living together	1.66±1.5
Patients changed dietary habit during the pandemic	36

**Table 2:** Changes in dietary habits and laboratory data before and during COVID-19 CRP: C-reactive protein; HDL-C: High-density lipoprotein cholesterol; K: Potassium; LDL-C: Low-density lipoprotein cholesterol; P: Phosphorus; T-Cho: Total cholesterol; TG: Triglycerides; WBC: White blood cells

Variable (unit: reference value)	Before the pandemic (n=132)	During the pandemic (n=132)	p-value
Dietary habit (times/week)			
Eating in	16.25±6.0	16.38±6.0	0.43
Eating takeout	3.16±5.1	3.20±5.0	0.83
Eating out	0.79±1.82	0.38±1.24	<0.01
Blood sampling data			
WBC (μL:3,100-8,400)	5,131±1,928	5,212±1,910	0.70
CRP (mg/L:0.0-0.3)	0.37±0.67	0.43±0.89	0.19
Hemoglobin (g/dL: 11.4-16.6)	10.73±1.53	10.59±1.36	0.46
P (mg/L:2.5-4.5)	5.03±1.04	5.13±1.15	0.44
K (mEq/L:3.5-5.0)	4.73±0.54	4.80±0.74	0.56
TG (mg/dl:20-149)	108.8±73.0	102.2±71.2	0.35
T-Cho (mg/dl:140-199)	147.8±30.2	147.1±33.3	0.66
HDL-C (mg/dl:40-96)	41.4±13.0	48.2±14.6	<0.01
LDL-C (mg/dl:60-119)	80.5±23.3	79.5±27.1	0.72

23 males (28.8%) and 13 females (25.0%) changed their dietary habits during the pandemic period, and during the pandemic. The frequency of eating-in, eating -takeout, and eating-out per week was 16.38 ±6.0, 3.20±5.0, and 0.38±1.24, respectively. There was a significant decrease in the frequency of eating out during the COVID-19 pandemic compared with that before the pandemic (data not shown).

**Figure 1:**
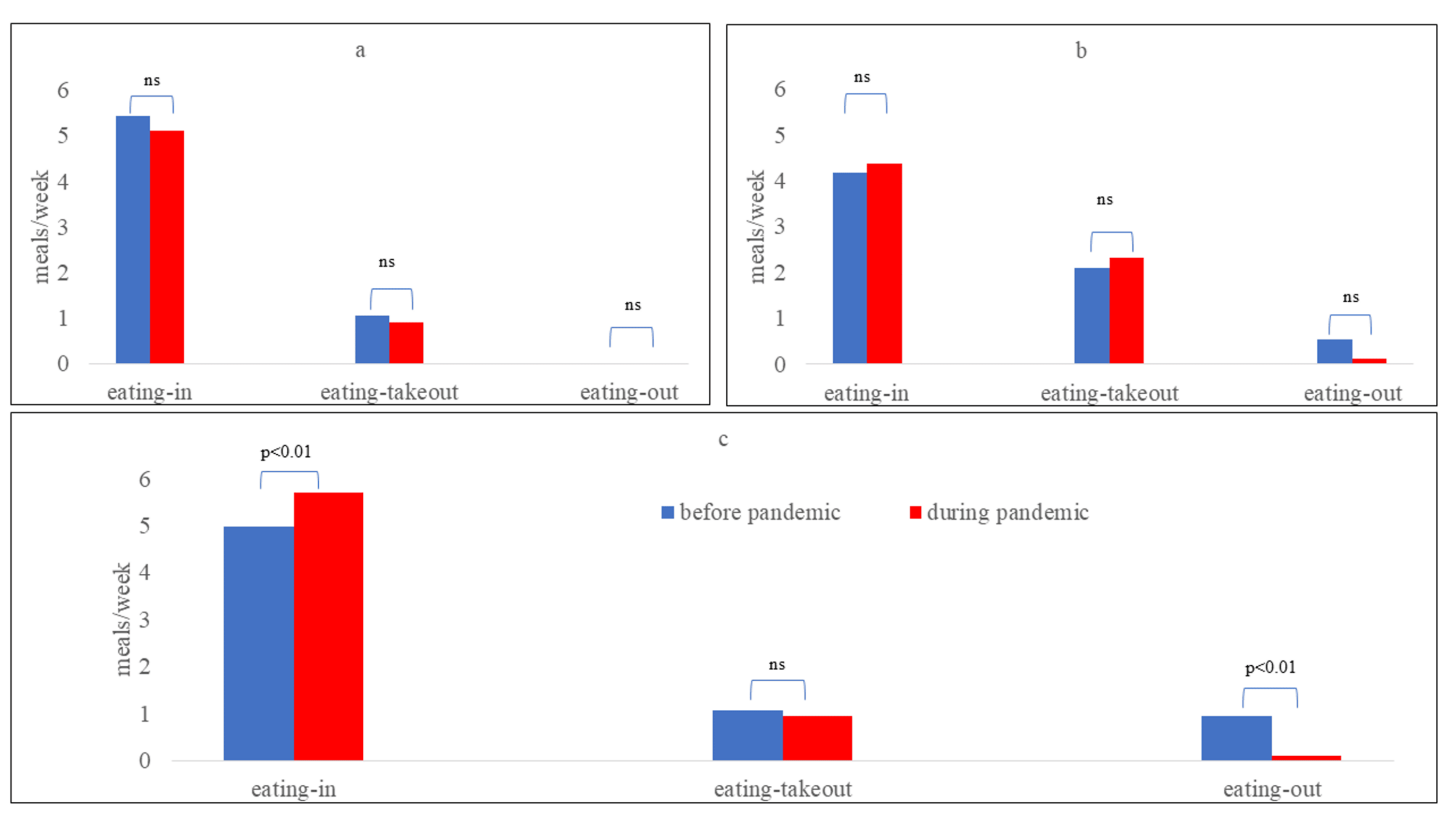
Comparison of approximate frequencies of each dietary habit (eating in, eating takeout, and eating out) at breakfast (a), lunch (b), and dinner (c), before and during the COVID-19 pandemic. Statistical comparisons were made using paired-samples t-test, with p<0.05 considered significant.

There was no significant difference before and during the COVID-19 pandemic in breakfast and lunch (Figure 1a, 1b), but the number of eating-out instances significantly decreased, and the number of eating-in instances increased for dinner during COVID-19 (Figure 1c). 

There were significant differences in HDL-C levels before and during the pandemic; however, they were within normal limits (Table 2).

At the beginning of this study, none of the patients had COVID-19. During the observation period, 35 patients were infected with COVID-19, all in the 7th and 8th Japanese waves of COVID-19, with household infection as the primary source. All patients infected with COVID-19 recovered well (Table 3). 

**Table 3:** Characteristics and comparison between patients with and without COVID-19 CRP: C-reactive protein; HD: Hemodialysis; HDL-C: High-density lipoprotein cholesterol; K: Potassium; LDL-C: Low-density lipoprotein cholesterol; P: Phosphorus; T-Cho: Total cholesterol; TG: Triglycerides; WBC: White blood cells

Variable (unit: reference value)	COVID-19(-) (n=97)	COVID-19(+) (n=35)	p-value
Male / Female	58 / 39	22 / 13	0.84†
Age (years）	70.4±11.6	73.8±10.7	0.14
Duration on HD (months)	111.6±79.4	95.6±96.4	0.34
Patients with diabetes	43	15	1†
Number of COVID-19 vaccination doses	2.94±0.43	2.74±0.85	0.08
Number of people living together	1.65 ±1.62	1.69 ±1.32	0.91
Patients changed dietary habit during pandemic	27	9	1†
Dietary habit (times/week)			
Eating in	15.7±6.4	18.2±4.2	0.03
Eating takeout	3.75±5.4	1.66±3.0	0.03
Eating out	0.39±1.25	0.34±1.23	0.86
Blood sampling data			
WBC (μL:3,100-8,400)	5,337±1,997	4,869±1,620	0.22
CRP (mg/L:0.0-0.3)	0.48±1.01	0.28±0.37	0.26
Hemoglobin (g/dL: 11.4-16.6)	10.63±1.44	10.48±1.10	0.56
P (mg/L:2.5-4.5)	5.14±1.10	5.09±1.31	0.83
K (mEq/L:3.5-5.0)	4.83±0.76	4.71±0.70	0.43
TG (mg/dl:20-149)	104.7±76.3	95.0±54.9	0.49
T-Cho (mg/dl:140-199)	150.3±33.7	138.5±31.1	0.07
HDL-C (mg/dl:40-96)	49.0±15.4	45.9±12.0	0.28
LDL-C (mg/dl:60-119)	81.5±27.0	74.2±27.1	0.17

The COVID-19-infected group had significantly higher frequencies of eating in and lower frequencies of eating takeout than the non-COVID-19 group.

Single and multivariate logistic regression analysis showed that only eating takeout was associated with a lower risk of COVID-19, with an adjusted OR of 0.889 (95%CI: 0.794-0.996, p=0.042) (Table 4).

**Table 4:** Univariate and multivariate regression analyses between COVID-19 and clinical laboratory parameters CI: Confidence interval; CRP: C-reactive protein; HD: Hemodialysis; HDL-C: High-density lipoprotein cholesterol; K: Potassium; LDL-C: Low-density lipoprotein cholesterol; OR: Odds ratio; P: Phosphorus; T-Cho: Total cholesterol; TG: Triglycerides; WBC: White blood cells

Univariate regression analysis			
Explanatory variable	OR	95% CI	P value
Male / Female	1.14	0.51 to 2.52	0.75
Age (years）	1.03	0.99 to 1.07	0.14
Duration on HD (months)	1	0.99 to 1.00	0.34
Patients with diabetes	0.94	0.43 to 2.05	0.88
Number of COVID-19 vaccination doses	0.61	0.33 to 1.12	0.11
Number of people living together	1.02	0.79 to 1.30	0.91
Patients changed dietary habit during pandemic	0.9	0.37 to 2.16	0.81
Dietary habit (times/week)			
Eating in	1.09	1.00 to 1.19	0.04
Eating takeout	0.89	0.79 to 0.99	0.04
Eating out	0.97	0.70 to 1.34	0.86
Blood sampling data			
WBC (μL:3,100-8,400)	0.87	0.69 to 1.09	0.22
CRP (mg/L:0.0-0.3)	0.67	0.32 to 1.40	0.28
Hemoglobin (g/dL: 11.4-16.6)	0.92	0.69 to 1.23	0.56
P (mg/L:2.5-4.5)	0.96	0.69 to 1.35	0.83
K (mEq/L:3.5-5.0)	0.81	0.48 to 1.37	0.43
TG (mg/dl:20-149)	0.99	0.99 to 1.00	0.49
T-Cho (mg/dl:140-199)	0.99	0.98 to 1.00	0.08
HDL-C (mg/dl:40-96)	0.99	0.96 to 1.01	0.28
LDL-C (mg/dl:60-119)	0.99	0.97 to 1.00	0.18
Multivariate regression analysis			
Eating in	1.04	0.89 to 1.21	0.62
Eating takeout	0.89	0.79 to 0.99	0.04
T-Cho (mg/dl:140-199)	0.99	0.98 to 1.00	0.69

## Discussion

The following two findings were obtained in this study. First, we investigated changes in dietary habits among MHPs, and 36 out of 132 (27%) MHPs changed their dietary habits. Consumption patterns for breakfast and lunch remained unchanged; however, for dinner, there was a significant decrease in eating out and an equivalent increase in eating in during the pandemic. Our findings are consistent with those of previous studies in non-dialysis patients, which also showed a decrease in the frequency of eating out and an increase in eating in [16,17]. Research on the dietary habits of MHPs is scarce. These dietary habits changes may be the effect of voluntary restrictions on outings issued by the Japanese government as a measure to prevent COVID-19. This study is the first to investigate the changes in dietary habits of MHPs in Japan. Second, our results suggest that eating takeout may help prevent COVID-19. Washing hands at least five times a day, wearing masks at home, eating alone or in silence, and using temporal and spatial isolation measures can effectively minimize the spread of COVID-19 infection in the home [15]. The frequency of eating takeout, in addition to eating alone and in silence, results in temporal and spatial isolation, which is believed to be associated with the prevention of COVID-19. Our results suggest that the person preparing the meal may be a more important factor than where the meal is eaten when the main route of infection is household transmission.

The main routes of transmission of COVID-19 are droplets, aerosols, and direct contact [18]. Therefore, the most appropriate means of protection is the use of masks and frequent hand washing with disinfectants. Minimizing human interactions is critical to preventing transmission. In particular, people should avoid sharing meals [9]. If a person with COVID-19 helps prepare food for his or her family, there is an increased likelihood of spreading the SARS-CoV-2 virus if he or she inadvertently mishandles the food or if contaminated respiratory droplets come into contact with the food or equipment [19]. This can lead to disease transmission among family members. Similarly, sharing dishes or plates among family members during meals increases the likelihood of cross-contamination. Therefore, it is important to avoid sharing meals with family members [15]. Bi et al. found that the likelihood of infection was greater among household contacts than among close contacts [20]. Our results suggest that preventing infection within a household is critical, as the group that had more shared meals, which is often associated with less household contact, had a lower incidence of COVID-19.

To date, there have been no papers showing an association between the number of people living together and COVID-19. In this study, we found no association between the number of people living together and COVID-19. Unlike healthy people, dialysis patients must undergo dialysis three times a week. During dialysis, it is often difficult to isolate patients temporally and spatially, which increases the possibility of infection not only in the home but also in the facility, which may explain why there was no association between the number of people living together and COVID-19.

This study has several limitations. First, it was a retrospective cohort study, and the number of participants was limited because all participants were from a single institution. Second, it was not possible to comprehensively examine all factors associated with changes in dietary habits. Third, the participants' responses regarding changes in dietary habits were self-reported and dependent on participants' recall.

## Conclusions

This study is the first to examine the relationship between dietary habits status and COVID-19 in MHPs, suggesting that for a disease such as COVID-19, which can be spread through food, in addition to where the food is eaten, who prepares it may also be relevant to infection prevention. In the future, MHPs are likely to encounter more unknown infections. The results of this study are expected to be useful for future prevention efforts.
